# Optical Properties of Electrically Tunable Two-Dimensional Photonic Lattice Structures Formed in a Holographic Polymer-Dispersed Liquid Crystal Film: Analysis and Experiment^†^

**DOI:** 10.3390/ma7053677

**Published:** 2014-05-07

**Authors:** Mayu Miki, Ryuichiro Ohira, Yasuo Tomita

**Affiliations:** Department of Engineering Science, University of Electro-Communications, 1-5-1 Chofugaoka, Chofu, Tokyo 182-8585, Japan; E-Mails: mayu_miki@ot.olympus.co.jp (M.M.); ryuichiro.ohira@jp.sony.com (R.O.)

**Keywords:** photonic crystal, holographic polymer-dispersed liquid crystal, photopolymer, liquid crystal, holographic polymerization

## Abstract

We report on theoretical and experimental investigations of optical wave propagations in two-dimensional photonic lattice structures formed in a holographic polymer-dispersed liquid crystal (HPDLC) film. In the theoretical analysis we employed the 2 × 2 matrix formulation and the statistical thermodynamics model to analyze the formation of anisotropic photonic lattice structures by holographic polymerization. The influence of multiple reflections inside an HPDLC film on the formed refractive index distribution was taken into account in the analysis. In the experiment we fabricated two-dimensional photonic lattice structures in an HPDLC film under three-beam interference holographic polymerization and performed optical measurements of spectral transmittances and wavelength dispersion. We also demonstrated the electrical control capability of the fabricated photonic lattice structure and its dependence on incident wave polarization. These measured results were compared with the calculated ones by means of photonic band and beam propagation calculations.

## 1. Introduction

Electro-optic photopolymer-liquid crystal (LC) composites known as holographic polymer-dispersed liquid crystals (HPDLCs) consist of LCs doped into photopolymer followed by holographic polymerization that represents a fast and relatively simple way of fabricating Bragg grating structures [1–4]. Because of the extremely large electro-optic response and the large optical anisotropy of LCs, HPDLCs have shown promises for electrically switchable Bragg grating devices including optical beam switching/control devices, lasers, optical memory and photonic crystals (PhCs) [5–9]. A different type of HPDLCs, polymer liquid-crystal polymer slices (POLICRYPS) or polymer liquid crystal polymer holograms electrically manageable (POLIPHEM) structures that can be fabricated above the LC nematic-isotropic transition temperature, has also been developed [10,11]. Because of their low light scattering properties as compared with HPDLCs possessing LC droplets they have also been used for various photonic applications [12–14]. Among reported device applications PhCs [15–18] possessing dielectric periodic structures in 2D and 3D space have been of great interest since they exhibit photonic band gaps (PBGs) in the optical spectrum. Such PBGs provide the strong control of light propagation in such a way that optical waves at certain wavelengths are either inhibited or confined in PhCs. The fabrication of PhCs using inorganic materials has already reached a mature state of evolution [17,19–22]. Inorganic semiconductor materials particularly possess high refractive indices and low absorption in the near-infrared spectral region, potentially exhibiting full PBGs. However, they typically lack the ease of active modification of PBGs for electrical or optical control of propagating light in PhCs, which are prerequisites for many photonic device elements. On the other hand, PhCs with LC-doped organic materials such as HPDLCs and POLICRYPS/POLIPHEM have the possibility of electrically tuning PBGs and therefore are interesting and viable candidates for tunable PhCs, although they do not possess a full potential PBGs owing to their much lower refractive index contrast than those with inorganic materials. So far, various organic PhC structures with HPDLCs have been realized by holographic polymerization [3,4] for electrical switching and lasing actions [23–32]. Moreover, interesting observations of strong wavelength dispersion and unusual refraction from a 2D honeycomb PhC fabricated by three-beam interference holographic polymerization in HPDLCs have been reported [33,34]. It was found that refraction angles due to the wavelength dispersion were strongly dependent on incident light wavelengths and angles. However, the electrical control capability with such a 2D honeycomb PhC has not been fully explored yet. In addition, it is an open question how PhC structures and their optical properties are altered with holographic polymerization when non-absorptive but reflective indium-tin oxide (ITO) electrodes on both sides of an HPDLC film exist for the electrical control. The 2D honeycomb photonic lattice structure is also interesting from a viewpoint of an optical analogue of graphene, a monolayer of graphite, in solid-state physics [35,36], by which new optical phenomena have been reported [37–42].

In this paper we describe theoretical and experimental investigations of the optical properties of electrically controllable 2D honeycomb (graphene)-type PhCs with HPDLCs. We employ the 2 × 2 matrix formulation [43] and the statistical thermodynamic model [44,45] to calculate light-intensity interference distributions and the corresponding anisotropic refractive index distributions in an HPDLC film under three-beam interference holographic exposure. The effect of multiple reflections recorded in an HPDLC film sandwiched between ITO-electrode loaded glass substrates on the formed interference pattern and the refractive index distribution is taken into account in the theoretical analysis. Experimental results of strong polarization-dependent spectral transmittances and wavelength-dependent refraction as well as their electrical control are also presented and are compared with the theoretical calculation.

## 2. Theoretical

### 2.1. Transfer Matrix Formulation

**Figure 1.**
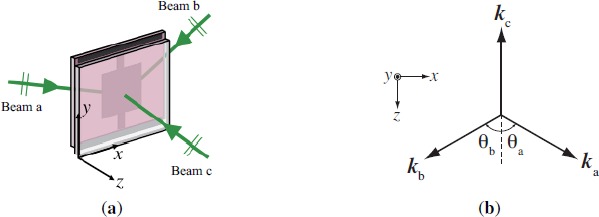
(**a**) Configuration of three-beam interference holographic polymerization and (**b**) its wave number (k-space) representation.

Let us consider holographic polymerization of a photosensitive film in a sample cell by mutually coherent three plane-wave beams at co-polarizations as shown in Figure 1. In our case the sample cell contains a thin film of HPDLC mixture sandwiched between two coplanar glass substrates on which non-absorptive but reflective ITO electrodes are deposited for an electric field application to the HPDLC film. In order to take the effect of multiple reflections between two ITO electrodes on the light-intensity interference distribution into account, we employ the 2 × 2 matrix formulation for isotropic layered media [43]. For the sake of simplicity we assume that nematic LCs mixed with host monomer were randomly oriented (*i.e.*, optically isotropic) before and during curing. This assumption may not be exactly correct in the LC-rich regions where LCs may undergo the nematic ordering along a preferred direction so that the HPDLC film becomes optically anisotropic. This happens when the volume fraction of LCs diffusing into the dark illuminated regions exceeds 0.8~0.9 during curing [46]. However, it would be legitimate to use the 2 × 2 matrix formulation in our analysis since the nematic reorientation process substantively occurs in the last half of the whole curing period where the conversion of monomer to polymer has substantively exceeded 50% [46].

**Figure 2.**
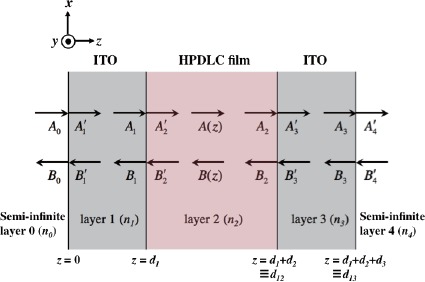
Three-layer system for the analysis.

Figure 2 shows a three-layer system sandwiched between two glass substrates that are treated as semi-infinite layers, where the *x*–*z* plane forms the plane of incidence as shown in Figure 1. In Figure 2 *A*(*z*) and *B*(*z*) correspond to amplitudes of right- and left-traveling waves, respectively, along the *z* direction in each layer.

Also, *A*_ℓ−1_ and ${B^{\prime }}_{\text{ℓ}}$ ( ${A^{\prime }}_{\text{ℓ}}$ and *B*_ℓ−1_) of right- and left-traveling waves denote amplitudes incident on (transmitted through) the interface between the (*ℓ* − 1)th and the *ℓ*th layers, respectively. Therefore, the following boundary conditions are given:

$$\begin{array}{llll} A_{0}=A(0^{-}), & B_{0}=B(0^{-}), & {A^{\prime }}_{1}=A(0^{+}), & {B^{\prime }}_{1}=B(0^{+}),\\ A_{1}=A(d_{1}^{-}), & B_{1}=B(d_{1}^{-}), & {A^{\prime }}_{2}=A(d_{1}^{+}), & {B^{\prime }}_{2}=B(d_{1}^{+}),\\ A_{2}=A(d_{12}^{-}), & B_{2}=B(d_{12}^{-}), & {A^{\prime }}_{3}=A(d_{12}^{+}), & {B^{\prime }}_{3}=B(d_{12}^{+}),\\ A_{3}=A(d_{13}^{-}), & B_{3}=B(d_{13}^{-}), & {A^{\prime }}_{4}=A(d_{13}^{+}), & {B^{\prime }}_{4}=B(d_{13}^{+}), \end{array}$$ (1)

where *z*^−^ (*z*^+^) denotes the left (right) side of the interface at *z*, and *d*_12_ and *d*_13_ stand for *d*_1_ + *d*_2_ and *d*_1_ + *d*_2_ + *d*_3_, respectively. Using the 2 × 2 matrix formulation, we can relate the amplitudes *A*_0_ and *B*_0_ to the amplitudes ${A^{\prime }}_{4}$ and ${B^{\prime }}_{4}$ as

$$\left[\begin{array}{l} A_{0}\\ B_{0} \end{array}\right]=M_{1}P_{2}M_{3}\left[\begin{array}{l} {A^{\prime }}_{4}\\ {B^{\prime }}_{4} \end{array}\right].$$ (2)

In Equation (2) *P*_2_ is a propagation matrix that accounts for the propagation through the bulk of the 2nd layer (*i.e.*, the HPDLC film). Its general form for the *l*th layer (*l* = 1, 2, 3) is given by

$$P_{l}=\left[\begin{array}{ll} e^{i\phi l} & 0\\ 0 & e^{-i\phi l} \end{array}\right],$$ (3)

where *ϕ_l_* is a phase shift for the *l*th layer of thickness *d_l_* and is given by *k_lz_d_l_*. Here *k_lz_* is the *z* component of the wavenumber *k_l_* given by

$$k_{lz}=n_{l}\frac{2\pi }{\lambda }\text{cos}\theta_{l},$$ (4)

where *n_l_* is the refractive index of the *l*th layer, λ is a wavelength in vacuum, and *θ_l_* is the ray angle with respect to the *z* axis in the *l*th layer. In Equation (2) a 2 *×* 2 transfer matrix *M_l_* for the *l*th layer (*l* = 1, 3) is given by [43]

$$M_{l}=\left[\begin{array}{ll} {\left(m_{l}\right)}_{11} & {\left(m_{l}\right)}_{12}\\ {\left(m_{l}\right)}_{21} & {\left(m_{l}\right)}_{22} \end{array}\right]$$ (5)

for s waves with

$$\begin{array}{l} {\left(m_{l}\right)}_{11}=\frac{1}{2}\left(1+\frac{n_{l+1}\text{cos}\theta_{l+1}}{n_{l-1}\text{ cos}\theta_{l-1}}\right)\text{cos}\phi_{l}+\frac{i}{2}\left(\frac{n_{l}\text{cos}\theta_{l}}{n_{l-1}\text{cos}\theta_{l-1}}+\frac{n_{l+1}\text{cos}\theta_{l+1}}{n_{l}\text{cos}\theta_{l}}\right)\text{sin}\phi_{l},\\ {\left(m_{l}\right)}_{12}=\frac{1}{2}\left(1-\frac{n_{l+1}\text{cos}\theta_{l+1}}{n_{l-1}\text{cos}\theta_{l-1}}\right)\text{cos}\phi_{l}+\frac{i}{2}\left(\frac{n_{l}\text{cos}\theta_{l}}{n_{l-1}\text{cos}\theta_{l-1}}-\frac{n_{l+1}\text{cos}\theta_{l+1}}{n_{l}\text{cos}\theta_{l}}\right)\text{sin}\phi_{l},\\ {\left(m_{l}\right)}_{21}=\frac{1}{2}\left(1-\frac{n_{l+1}\text{cos}\theta_{l+1}}{n_{l-1}\text{cos}\theta_{l-1}}\right)\text{cos}\phi_{l}-\frac{i}{2}\left(\frac{n_{l}\text{cos}\theta_{l}}{n_{l-1}\text{cos}\theta_{l-1}}-\frac{n_{l+1}\text{cos}\theta_{l+1}}{n_{l}\text{cos}\theta_{l}}\right)\text{sin}\phi_{l},\\ {\left(m_{l}\right)}_{22}=\frac{1}{2}\left(1+\frac{n_{l+1}\text{cos}\theta_{l+1}}{n_{l-1}\text{cos}\theta_{l-1}}\right)\text{cos}\phi_{l}-\frac{i}{2}\left(\frac{n_{l}\text{cos}\theta_{l}}{n_{l-1}\text{cos}\theta_{l-1}}+\frac{n_{l+1}\text{cos}\theta_{l+1}}{n_{l}\text{cos}\theta_{l}}\right)\text{sin}\phi_{l}, \end{array}$$ (6)

and for p waves with

$$\begin{array}{l} {\left(m_{l}\right)}_{11}=\frac{1}{2}\left(\frac{\text{cos}\theta_{l+1}}{\text{cos}\theta_{l-1}}+\frac{n_{l+1}}{n_{l-1}}\right)\text{cos}\phi_{l}+\frac{i}{2}\left(\frac{n_{l}\text{cos}\theta_{l+1}}{n_{l-1}\text{cos}\theta_{l}}+\frac{n_{l+1}\text{cos}\theta_{l}}{n_{l}\text{cos}\theta_{l-1}}\right)\text{sin}\phi_{l},\\ {\left(m_{l}\right)}_{12}=\frac{1}{2}\left(\frac{\text{cos}\theta_{l+1}}{\text{cos}\theta_{l-1}}-\frac{n_{l+1}}{n_{l-1}}\right)\text{cos}\phi_{l}+\frac{i}{2}\left(\frac{n_{l}\text{cos}\theta_{l+1}}{n_{l-1}\text{cos}\theta_{l}}-\frac{n_{l+1}\text{cos}\theta_{l}}{n_{l}\text{cos}\theta_{l-1}}\right)\text{sin}\phi_{l},\\ {\left(m_{l}\right)}_{21}=\frac{1}{2}\left(\frac{\text{cos}\theta_{l+1}}{\text{cos}\theta_{l-1}}-\frac{n_{l+1}}{n_{l-1}}\right)\text{cos}\phi_{l}-\frac{i}{2}\left(\frac{n_{l}\text{cos}\theta_{l+1}}{n_{l-1}\text{cos}\theta_{l}}-\frac{n_{l+1}\text{cos}\theta_{l}}{n_{l}\text{cos}\theta_{l-1}}\right)\text{sin}\phi_{l},\\ {\left(m_{l}\right)}_{22}=\frac{1}{2}\left(\frac{\text{cos}\theta_{l+1}}{\text{cos}\theta_{l-1}}+\frac{n_{l+1}}{n_{l-1}}\right)\text{cos}\phi_{l}-\frac{i}{2}\left(\frac{n_{l}\text{cos}\theta_{l+1}}{n_{l-1}\text{cos}\theta_{l}}+\frac{n_{l}\text{cos}\theta_{l}}{n_{l-1}\text{cos}\theta_{l-1}}\right)\text{sin}\phi_{l}, \end{array}$$ (7)

The amplitudes *A*(*z*) and *B*(*z*) in *d*_1_ ≤ *z* ≤ *d*_12_ and the amplitudes ${A^{\prime }}_{4}$ and ${B^{\prime }}_{4}$ are related as

$$\left[\begin{array}{l} A\left(z\right)\\ B\left(z\right) \end{array}\right]=\left[\begin{array}{ll} \text{exp}\left[ik_{2z}\left(d_{12}-z\right)\right] & 0\\ 0 & \text{exp}\left[-ik_{2z}\left(d_{12}-z\right)\right] \end{array}\right]M_{3}\left[\begin{array}{l} {A^{\prime }}_{4}\\ {B^{\prime }}_{4} \end{array}\right].$$ (8)

Note that one can put ${B^{\prime }}_{4}=0$ for a right-traveling incident wave with the amplitude *A*_0_ (*i.e.*, there is no reflection in the semi-infinite 4th layer) since the glass substrate-air boundaries (the glass substrate-prism boundaries in our experiment as shown later) are assumed to be index matched. Then, we find from Equation (2) that the amplitude ${A^{\prime }}_{4}$ is given by

$${A^{\prime }}_{4}=\frac{1}{m_{11}}A_{0},$$ (9)

where *m_ij_* (*i, j* = 1, 2) is a matrix element of the 2 × 2 transfer matrix *M* for the three-layer system as defined by

$$M=M_{1}P_{2}M_{3}$$ (10)

Substituting either Equation (6) or Equation (7) with ${B^{\prime }}_{4}=0$ into Equation (5) and using Equation (3), we obtain

$$A(z)=\frac{{\left(m_{3}\right)}_{11}}{m_{11}}\text{exp}\left[ik_{2z}(-z+d_{12})\right]A_{0}$$ (11)

And

$$B(z)=\frac{{\left(m_{3}\right)}_{21}}{m_{11}}\text{exp}\left[-ik_{2z}(-z+d_{12})\right]A_{0}$$ (12)

Thus, the resultant amplitude $E_{A_{0}}\left(x,z\right)$ in *d*_1_ *≤ z ≤ d*_12_ for an obliquely incident right-traveling plane wave having the amplitude *A*_0_(*x*) from the semi-infinite 0th layer is given by

$$E_{A_{0}}(x,z)=\frac{1}{m_{11}}\left[{\left(m_{3}\right)}_{11}\text{exp}[ik_{2z}(-z+d_{12})]+{\left(m_{3}\right)}_{21}\text{exp}[-ik_{2z}(-z+d_{12})]\right]A_{0}(x)$$ (13)

In the same way the resultant amplitude at *z*, $E_{{B^{\prime }}_{4}}\left(x,z\right)$ in *d*_1_ *≤ z ≤ d*_12_ for an obliquely incident left-traveling plane wave having the amplitude ${B^{\prime }}_{4}\left(x\right)$ from the semi-infinite 4th layer is given by

$$E_{{B^{\prime }}_{4}}(x,z)=\left\{\frac{1}{m_{11}}[-{\left(m_{3}\right)}_{11}m_{12}+{\left(m_{3}\right)}_{12}m_{11}]\text{exp}[ik_{2z}(-z+d_{12})]+[-{\left(m_{3}\right)}_{21}m_{12}+{\left(m_{3}\right)}_{22}m_{11}]\text{exp}[-ik_{2z}(-z+d_{12})]\right\}{B^{\prime }}_{4}(x).$$ (14)

Finally, we can calculate the 2D intensity distribution *I*(*x, z*) in *d*_1_ *≤ z ≤ d*_12_ as a result of the interference between three incident plane waves (two from the 0th layer and the other from the 4th layer), as given by

$$I(x,z)=|{\left[E_{A_{0}}(x,z)\right]}_{a}+{\left[E_{A_{0}}(x,z)\right]}_{b}+E_{{B^{\prime }}_{4}}(z){|}^{2}$$ (15)

where [*A*_0_(*x*)]*_a_* and [*A*_0_(*x*)]*_b_* are obliquely incident right-traveling plane waves at their incident ray angles *θ_a_* and *θ_b_*, respectively, in the 0th layer, and ${B^{\prime }}_{4}$ is one normally incident left-traveling plane wave (see Figure 1 and the experimental setup shown in Figure 7, in which amplitudes of the three incident plane waves with a unit amplitude are given by

$$\begin{array}{c} {\left[A_{0}(x)\right]}_{a}=\text{exp}(-ikn_{0}\text{sin}\theta_{a}x),\\ {\left[A_{0}(x)\right]}_{b}=\text{exp}(-{\text{ikn}}_{0}\text{sin}\theta_{b}x),\\ B^{\prime }{}_{4}=1. \end{array}$$ (16)

### 2.2. Light-Intensity Interference Distributions

In order to numerically evaluate the 2D intensity distribution *I*(*x, z*) given by Equation (15), we use the following numerical data: the refractive indices of glass substrates (the semi-infinite 0th and 4th layers) and non-absorptive but reflective ITO electrodes (the 1st and 3rd layers) are given by *n*_0_ = *n*_4_ = 1.52 and *n*_1_ = *n*_3_ = 1.95, respectively. The refractive index of the HPDLC film (an LC-monomer blend) is calculated in the following way: since LCs are assumed to be randomly distributed on average in a host material, its average refractive index *n_LC_* is given by $\sqrt{\left(2n_{o}^{2}+n_{e}^{2}\right)/3}$, where *n_o_* and *n_e_* are ordinary and extraordinary refractive indices of LC, respectively. For a nematic LC used in our experiment (see subsection 3.1) *n_LC_* is calculated to be 1.599 with *n_o_* = 1.529 and *n_e_* = 1.730. Using the Lorentz-Lorenz formula [47] with the HPDLC mixture of 40.8 vol.% nematic LC and 59.2 vol.% photopolymer blend (*n_polymer_*= 1.545), we found *n*_2_, the average refractive index of the HPDLC film (the 2nd layer, see Figure 2), to be 1.567.

**Figure 3.**
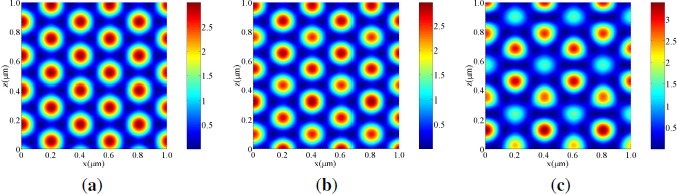
Calculated intensity distributions under s-polarized three-beam interference holographic exposure at a wavelength of 532 nm (**a**) in the free space and in the HPDLC film (**b**) without and (**c**) with ITO electrodes.

**Figure 4.**
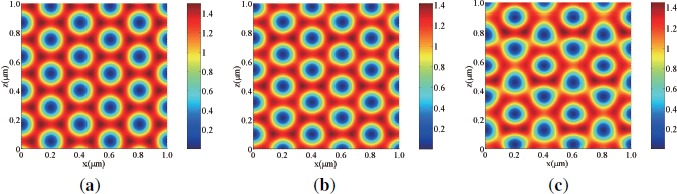
Calculated intensity distributions under p-polarized three-beam interference holographic exposure at a wavelength of 532 nm (**a**) in the free space and in the HPDLC film (**b**) without and (**c**) with ITO electrodes.

Figure 3(a) shows the calculated intensity distribution in the free space (*i.e.*, we set *n*_0_ = *n*_2_ = *n*_4_ = 1.567 and *d*_1_ = *d*_3_ = 0) by three s-polarized plane-wave interference polymerization at *θ_a_* = *−θ_b_* = 60° and at a wavelength of 532 nm. It can be seen that, as reported for a simple three-beam interference configuration [28,33], the intensity distribution exhibits the triangular lattice symmetry [17]. Figure 3(b) and Figure 3(c) show the calculated intensity distributions in the HPDLC film (*d*_2_ = 10 *μ*m) without (*i.e.*, *d*_1_ = *d*_3_ = 0) [Figure 3(b)] and with (*i.e.*, *d*_1_ = *d*_3_ = 25 nm) [Figure 3(c)] ITO electrodes by three s-polarized plane-wave interference polymerization. It can be seen that the intensity distributions are strongly influenced by multiple reflections between two ITO electrodes and that the perfect triangular lattice symmetry seen in the free space is broken as multiple reflections is stronger. Figure 4 shows the calculated intensity distributions in the free space [Figure 4(a)] and in the HPDLC film (*d*_2_ = 10 *μ*m) without [Figure 4(b)] and with [Figure 4(c)] ITO electrodes by three p-polarized plane-wave interference polymerization. It can be seen in [Figure 4(a)] that the 2D intensity distribution in the free space exhibits the same triangular lattice symmetry as the case of s waves but its contrast is lower and reversed. In order to explain this change, we consider the 2D intensity distribution *I*(*x, z*)[*≡ I*(r)] in the free space for the configuration of three-beam interference holographic polymerization (see Figure 1 with *a* = 1, *b* = 2 and *c* = 3) with their unit amplitudes as given by

$$I(r)=\sum_{m=1}^{3}\sum_{n=1}^{3}e_{m}\cdot e_{n}^{*}\text{exp}[-i(G_{mn}\cdot r+\phi_{mn})],$$ (17)

where e*_m_* is a complex eigen polarization vector for beam *m*, G*_mn_*(*≡* k*_m_ −* k*_n_*) is the reciprocal wave vector and *ϕ_mn_*(*≡ϕ_m_ − ϕ_n_*) is the relative phase between the constant phases of *ϕ_m_* and *ϕ_n_* for beams *m* and *n*, respectively. It is straightforward to show that for three s or p polarized beams at *θ*_1_ = *−θ*_2_ = 60*° I*(r) is given by

$$I(r)=\{\begin{array}{cc} 3+2[\text{cos}(G_{12}\cdot r+\phi_{12})+\text{cos}(G_{13}\cdot r+\phi_{13})+\text{cos}(G_{23}\cdot r+\phi_{23})], & \text{for}\;s\;\text{waves;}\\ 3-[\text{cos}(G_{12}\cdot r+\phi_{12})+\text{cos}(G_{13}\cdot r+\phi_{13})+\text{cos}(G_{23}\cdot r+\phi_{23})], & \text{for}\;p\;\text{waves.} \end{array}$$ (18)

Equation (18) shows that the 2D intensity distribution in the free space for p waves exhibits the same symmetry as the case of s waves but its contrast is lower and reversed, independently of relative phases between three beams. It can also be seen in Figure 4(b) and Figure 4(c) that multiple reflections tends to break the triangular lattice symmetry with different unit cell patterns from the case of s waves. Therefore, we expect that loading of non-absorptive but reflective ITO electrodes (for the electrical control purpose) strongly influences on the symmetry of PhC structures. In order to avoid this effect, one may need to form a dielectric matching layer on ITO electrodes.

### 2.3. Refractive Index Distributions and Numerical Method

We are now in a position to calculate 2D refractive index distributions in the HPDLC film with ITO electrodes by using the statistical thermodynamics model [44] with the help of the calculated 2D intensity distributions. The model employs the Flory-Huggins chemical potential, together with the photopolymerization-diffusion equation, for a three-component system (monomer, polymer and LC) to obtain 2D concentration distributions of the three components during and after photopolymerization. In this case we set the polymerization rate of LCs to be zero. We also assume the diffusion-dominant polymerization process and the same size of an LC molecule as that of monomer. Furthermore, we introduce the threshold LC volume fraction *ϕ_LC_|_th_* (=0.8*~*0.9) that determines whether LCs are either randomly oriented at the volume fraction of LCs *ϕ_LC_ < ϕ_LC_|_th_* or aligned by the nematic ordering along the *y* direction in high *ϕ_LC_* regions forming the cylinder-like LC channels (cavities) [25] at *ϕ_LC_* ≥ *ϕ_LC_|_th_*. This assumption is approximately justified by our experiments as described in subsection 4.2. Under this assumption the position-dependent refractive index of LCs for s (p) waves is given by the formula $\sqrt{\left(2n_{o}^{2}+n_{e}^{2}\right)/3}\left(\sqrt{\left(2n_{o}^{2}+n_{e}^{2}\right)/3}\right)$ at *ϕ_LC_* < *ϕ_LC_|_th_* and is given by *n_e_* (*n_o_*) at *ϕ_LC_* ≥ *ϕ_LC_|_th_*. We also assume that when an external electric field E*_ex_* (being higher than the threshold voltage [48]) is applied along the *z* direction, all LCs reorient along the *z* direction. In this way we can calculate 2D anisotropic refractive index distributions for s and p waves via the Lorentz-Lorenz formula with calculated concentrations and refractive indices of the formed polymer and LCs.

**Figure 5.**
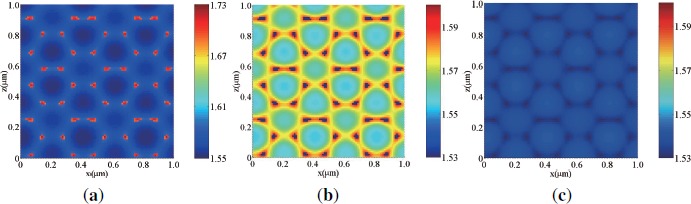
Calculated refractive index distributions by s-polarized three-beam interference holographic polymerization when no *E_ex_* is applied for (**a**) s (TM) and (**b**) p (TE) wave readout, and (**c**) when *E_ex_* is applied for any polarized wave. Note that the minimum and maximum values for the color bar levels in (**a**) are different from those in (**b**) and (**c**).

Figure 5 shows calculated 2D refractive index distributions by three s-polarized plane-wave interference polymerization when no *E_ex_* is applied for s [Figure 5(a)] and p [Figure 5(b)] waves, and when *E_ex_* is applied for any polarized wave [Figure 5(c)]. In this calculation we set *ϕ_LC_|_th_* to be 0.8. It can be seen that LC is rich in the dark illuminated regions as a result of the mutual diffusion of monomer and photo-insensitive species (*i.e.*, LCs) [4,45,49,50]. The refractive index of the HPDLC film is higher (lower) in the LC-rich regions than in the formed polymer-rich regions for s (p) waves since the refractive index of LCs is equal to *n_e_* (*n_o_*) at *ϕ_LC_* ≥ *ϕ_LC_|_th_*. It can also be seen that the formed 2D refractive index distributions for s and p wave readout exhibit complementary 2D honeycomb-like PhC structures each other although s wave readout probes higher contrast structure than p wave readout. The contrast reversal of the PhC structures between s and p wave readout is understandable because the refractive index at the lattice sites (*i.e.*, the lowest intensity regions) is *n_e_* (*n_o_*) for s (p) wave. When *E_ex_* is on, LCs reorient along the *z* direction. In this case both s and p waves sense only a difference in refractive index between *n_o_* (=1.53) and *n_polymer_* (=1.545), resulting in very low contrast PhC structure as shown in Figure 5(c).

**Figure 6.**
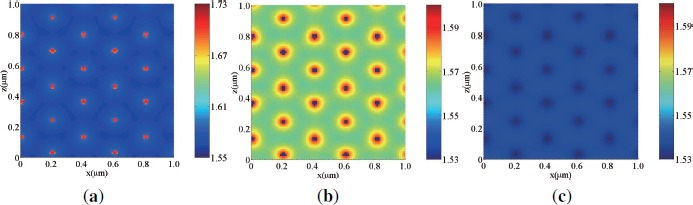
Calculated refractive index distributions by p-polarized three-beam interference holographic polymerization when no *E_ex_* is applied for (**a**) s (TM) and (**b**) p (TE) wave readout, and (**c**) when *E_ex_* is applied for any polarized wave. Note that the minimum and maximum values for the color bar levels in (**a**) are different from those in (**b**) and (**c**).

Figure 6 shows calculated 2D refractive index distributions by three p-polarized plane-wave interference polymerization when no *E_ex_* is applied for s [Figure 6(a)] and p [Figure 6(b)] waves, and when *E_ex_* is applied for any polarized wave [Figure 6(c)].

It can be seen that the 2D refractive index distributions for s [Figure 6(a)] and p [Figure 6(b)] waves exhibit complementary 2D triangular-like PhC structures since LCs migrates toward lower intensity regions that form a 2D triangular-like PhC structure as seen in Figure 4(c). When *E_ex_* is on, both s and p waves sense only a difference in refractive index between *n_o_* (=1.53) and *n_polymer_* (=1.545), resulting in very low contrast PhC structure as shown in Figure 6(c).

Our method of numerical analyses is as follows: numerical data of the calculated 2D refractive index distributions obtained by the method described so far were used with a commercially available numerical software package (CrystalWave, Photon Design) by which the PhC properties (*i.e.*, photonic band structures, anisotropic spectral transmittances and wavelength dispersion) were numerically analyzed with the built-in programs using the finite difference time domain (FDTD) method and the plane-wave expansion method [51].

## 3. Experimental

### 3.1. Sample Preparation and Three-Beam Interference Holographic Polymerization

**Figure 7.**
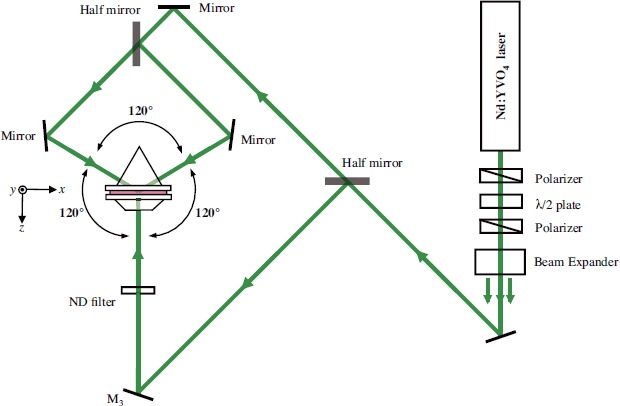
Experimental setup for three-beam interference holographic polymerization.

We prepared HPDLC syrup consisting of the 30 wt.% mixture of a nematic LC (TL203, Merck) with a monomer blend of 34 wt.% of multifunctional acrylate monomer (Ebecryl8301, Cytec), 11.5 wt.% of thiol-ene-based monomer (NOA65, Norland) and 18.3 wt.% *N*-vinyl pyrrolidone (Tokyo Chemical Industry). We also used 4 wt.% of octanoic acid (Tokyo Chemical Industry) that acted as surfactant. To sensitize in the green (532 nm), we employed 0.7 wt.% of Rose Bengal (Tokyo Chemical Industry) and 1.5 wt.% of *N*-phenyl-glycine (Tokyo Chemical Industry). The HPDLC syrup was injected by the capillary action into a sample cell consisting of two glass substrates with a 10 *μ*m-thick spacer. We note that the commercially available thiol-ene prepolymer NOA65 contains a trifunctional thiol and a tetrafunctional urethane allyl ether ene, exhibiting free radical mediated step-growth polymerizations [52,53]. It was reported that HPDLCs based on thiol-ene monomers gave smaller LC droplets and much lower light scattering than HPDLCs based on acrylate-based monomers capable of free radical mediated chain-growth polymerizations [53–55]. Indeed, we did not observe noticeable light scattering from our HPDLC samples during and after curing. Such a low light scattering property would come from the fact that the average size of the formed LC droplets was much smaller than a wavelength of recording beams (532 nm) in the microscopic morphology of our 10-*μ*m-thick HPDLC film samples as shown in the next section. Measured refractive indices of LC were *n_e_* = 1.730 and *n_o_* = 1.529 at extraordinary and ordinary polarizations, respectively, while those of the photopolymer blend were 1.503 and 1.545 in the liquid and solid phases, respectively. All these refractive indices were measured by an Abbe refractometer (DR-M2, ATAGO) at a wavelength of 546 nm. ITO electrodes were deposited on two glass substrates for an application of an electric field between the two glass substrates as shown in Figure 1. An experimental setup for holographic polymerization and transmission measurements is shown in Figure 7. A coherent, linearly polarized and expanded beams from a diode pumped frequency-doubled Nd:YVO_4_ laser operating at a wavelength of 532 nm was divided into two mutually coherent beams with equal intensities by a half mirror. One of the two beams was normally incident onto the sample through a neutral density (ND) filter and a trapezoidal BK7 prism (*n* = 1.52), whereas the other beam was divided into two equal-intensity beams that were incident onto the sample at the bisector angle of 120° via an equilateral triangular BK7 prism (*n* = 1.52). Index matching oil (*n* = 1.52) was filled in between these prisms and the sample cell to avoid unwanted refraction and multiple reflections of incident beams between the prisms and the HPDLC film sample cell. The three coplanar beams at equal intensities (67 mW/cm^2^) and at co-polarizations (three s or p waves) interfered with one another, giving a 2D light-intensity distribution in the *x*–*z* plane. Such holographic exposure created a 2D refractive index distribution in the HPDLC film as described in Subsection 2.3. After 20-minutes holographic exposure the HPDLC film was post-cured with an ultraviolet LED light source operating at a wavelength of 365 nm for 30 minutes to ensure that remaining monomer, if any, was consumed completely. Although we employed a conventional three-beam holographic setup [56,57] without any particular phase stabilization control [58,59], any nonlinear effect such as wave mixing between interfering beams during curing [60] would not play a role in our fabrication process. This is so because good agreement is seen between the morphology of our fabricated PhC structures and the calculated ones as will be seen in Subsection 4.1.

### 3.2. Measurements of Spectral Transmittance and Wavelength Dispersion

Spectral transmittances of the recorded HPDLC film were measured by use of a white light source (LS-11-LL, Ocean Optics) and a fiber-coupled spectrometer (ORIEL FICS, ORIEL). The collimated and linearly polarized white light was normally incident on the patterned HPDLC film along the *z* direction. We also employed linearly polarized laser beams operating at various wavelengths (404 nm, 532 nm and 632.8 nm) to examine the wavelength dispersion characteristics of the patterned HPDLC film at various incident angles. In these measurements two semi-spherical BK7 prisms (*n* = 1.52) contacted to both surfaces of the recorded HPDLC film sample cell with the index-matching oil to avoid unwanted reflections and refraction at the glass substrate-air boundaries. In order to investigate a dependence of spectral transmittances on an externally applied electric field and the wavelength dispersion characteristics, we employed a bipolar 2 kHz square-wave voltage applied to the two ITO electrodes.

## 4. Results and Discussion

### 4.1. Morphology of Fabricated Photonic Crystal Structures

**Figure 8.**
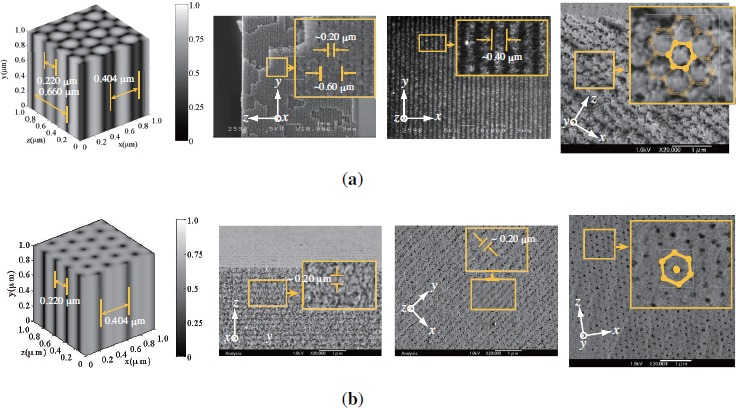
3D polymer structures for HPDLC films under three-beam interference holographic polymerization by using three (**a**) s- and (**b**) p-polarized plane waves. Calculated 3D polymer distributions in volume fraction, cross sectional SEM images in the *y*–*z*, *x*–*y* and *x*–*z* planes are shown in order from the left in (**a**) and (**b**).

We examined the morphology of holographically patterned PhC structures in HPDLC films by removing LC compound from PhC lattice sites. This was done by dipping cured HPDLC films into methanol and by observing the polymer structures in various cross sectional planes by means of a scanning electron microscopy (SEM). Figure 8 shows cross sectional SEM images of the polymer structures in the *y*–*z*, *x*–*y* and *x*–*z* planes for HPDLC films under three-beam interference polymerization by using three s-polarized [Figure 8(a)] and p-polarized [Figure 8(b)] plane waves. The calculated 3D polymer concentration distributions in volume fraction are also shown at the leftmost positions of Figure 8(a) and Figure 8(b), where bright portions correspond the polymer-rich (LC-poor) regions and dark portions correspond to polymer-poor (LC-rich) regions in which LCs were washed away after the methanol treatment. It can be seen that the observed SEM images are in good agreement with the calculated polymer structures in terms of their morphology and sizes when polymerization shrinkage predominantly taken place along the thickness (*z*) direction is taken into account. More specifically, calculated and measured periods of the polymer structures along the *z* direction are (0.220 and 0.660 *μ*m) and (*∼*0.20 and *∼*0.60 *μ*m), and these differences are of the order of polymerization shrinkage (approximately a few %, typical for multifunctional acrylates and our monomer blend).

**Figure 9.**
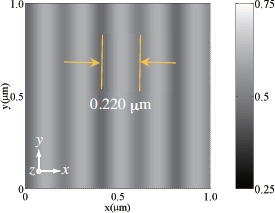
Calculated cross section of the polymer concentration distribution in the *x*–*y* plane at *z* = 0.078 *μ*m prepared by p-polarized three-beam interference holographic polymerization.

We also note that the observed 3D polymer structure created by s-polarized three-beam interference polymerization [Figure 8(b)] possess the grating period of *∼*0.20 *μ*m along the *x* direction in the *x*–*y* plane, which is shorter than the calculated one of 0.404 *μ*m at *z* = 0. This discrepancy can be understood by examining the calculated polymer structure in the *x*–*y* plane at *z* = 0.078 *μ*m, as shown in Figure 9. It can be seen that the adjacent spacing between low and high concentration modulations [see the cross section along the *x* direction 0.078 at *z* = 0.078 *μ*m in Figure 6(b)] is 0.202 *μ*m, in good agreement with the measured value. In what follows we consider the case of s-polarized three-beam interference holographic polymerization since, as shown later, no noticeable wavelength dispersion was observed with three p waves.

### 4.2. Spectral Transmittance

**Figure 10.**
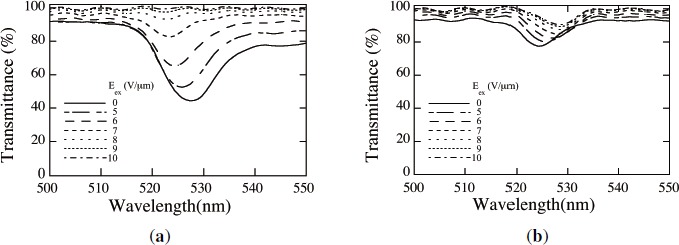
Measured spectral transmittances of of an HPDLC film recorded by three s-polarized plane-wave interference holographic polymerization and readout by normally incident (**a**) s and (**b**) p waves for different values of E*_ex_*.

Figure 10 shows measured spectral transmittances of an HPDLC film recorded by three s-polarized plane-wave interference holographic polymerization and readout by normally incident s [Figure 10(a)] and p [Figure 10(b)] waves for different values of E*_ex_*. It can be seen that the transmittance spectra have a strong readout-polarization dependence. The observed Bragg reflection (*i.e.*, the transmittance minimum) for s waves is much significant than that for p waves in the absence of E*_ex_*. This result indicates that, as is consistent with our assumption made in our theoretical analysis, nematically ordered LCs in the regions above *ϕ_LC_|_th_* predominantly orient along the *y* direction in the absence of E*_ex_*, giving the high contrast PhC structure for s wave readout as seen in Figure 5(a). The observed Bragg reflection for p wave readout would have contribution from the *x*-direction component of LC directors due to the statistical distributions of LCs in *x*–*z* plane as a result of their thermal fluctuations [61]. It can also be seen that the observed Bragg reflections for both s and p wave readout in the absence of E*_ex_* take place at the Bragg wavelengths λ_Bragg_ of 528 and 525 nm, respectively, that are shorter than the recording wavelength of 532 nm. We speculate that such blue shifts are caused by polymerization shrinkage of the order of a few % as mentioned earlier. The asymmetry in the spectral transmittance profiles, prominent for s wave readout, may also be caused by the distortion of the recorded 2D PhC structure due to the polymerization shrinkage and to the residual background absorption from Rose Bengal dyes that tend to orient along LC directors. We also see that the application of E*_ex_* higher than 9 V/*μ*m diminishes Bragg reflection for s wave readout. This is so because most of nematically ordered LCs reorient along the *z* direction at E*_ex_* higher than 9 V/*μ*m so that the PhC structure tends to disappear as seen in Figure 5(c). The decreasing trend of λ_Bragg_ for s wave readout with an increase in E*_ex_* can also be explained by the reorientation of LCs so that the average refractive index of the recorded 2D PhC structure decreases. On the other hand, it can be seen in Figure 10(b) that the application of E*_ex_* higher than 9 V/*μ*m does not sufficiently diminishes the PBG structure for p wave readout. We speculate that while most of nematically ordered LCs reorient under E*_ex_* along the *z* direction, randomly oriented LCs embedded in the polymer-rich regions are not completely reorient along the *z* direction. This trend would result in non-negligible refractive index modulation unlike our theoretical model that assumes the complete reorientation of all LCs under E*_ex_* along the *z* direction. The increasing trend of λ_Bragg_ for p wave readout with an increase in E*_ex_* suggests an increase in the average refractive index of the recorded 2D PhC structure, which may be explained by an increase in the statistically distributed *x* component of nematically ordered LCs with increasing E*_ex_*.

**Figure 11.**
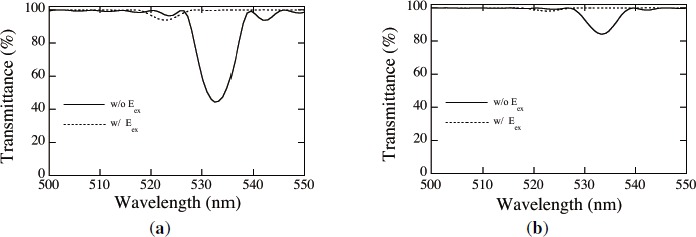
Calculated spectral transmittances of an HPDLC film recorded by three p-polarized plane-wave interference holographic polymerization and readout by normally incident (**a**) s and (**b**) p waves without and with E*_ex_*.

Figure 11 shows calculated spectral transmittances of HPDLC films recorded by three p-polarized plane-wave interference holographic polymerization and readout by normally incident s [Figure 10(a)] and p [Figure 10(b)] waves without and with E*_ex_*. We chose *ϕ_LC_|_th_* = 0.9 that reproduced similar Bragg minimum transmittances without E*_ex_* for s and p wave readout to the measured ones shown in Figure 10.

The calculated Bragg transmittance without E*_ex_* for p wave readout is much higher than that for s wave readout, consistent with the measured results. The decreasing trend of λ_Bragg_ in the presence of E*_ex_* for s wave readout due to a decrease in the average refractive index is also consistent with the measured result as mentioned above. Therefore, we consider that our simplified assumption of the director orientation of nematically ordered LCs above *ϕ_LC_|_th_* along the *y* direction in the absence of E*_ex_* is adequate. The disappearance of Bragg transmittance in the presence of E*_ex_* for p wave, as seen in Figure 11(b), results from the assumption of our theoretical model that all LCs reorient along the *z* direction as described in Subsection 2.3. The discrepancy in an E*_ex_*-induced transmittance change between theoretical and experimental results may be explained by, as described above, the insufficient reorientation of randomly oriented LCs embedded in the polymer-rich regions under E*_ex_* along the *z* direction, which maintains the refractive index contrast of the 2D PhC structure to some extent. The discrepancy in the E*_ex_*-induced shift of λ_Bragg_ for p wave readout between theoretical and experimental results may be explained by an increase in the statistically distributed *x* component of nematically ordered LCs with increasing E*_ex_*, as mentioned above. Further investigation is necessary to clarify the discrepancy.

### 4.3. Wavelength Dispersion Characteristics

**Figure 12.**
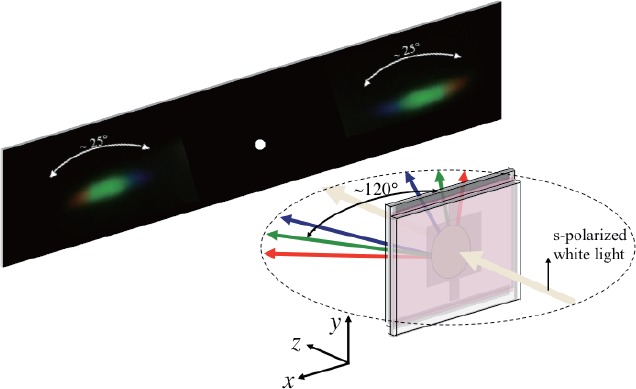
Observed wavelength dispersion pattern from an HPDLC film recorded by three s-polarized plane-wave interference holographic polymerization and readout by normally incident s-polarized white light in the absence of E*_ex_*.

We examined the wavelength dispersion characteristics of our 2D honeycomb-like PhC and its electrical control. Figure 12 illustrates the result for an HPDLC film recorded by three s-polarized plane-wave interference holographic polymerization and readout by normally incident s-polarized white light in the absence of E*_ex_*. We observed the symmetric wavelength dispersion from the blue to the red with the full dispersion-angle width of approximately 25°. The observed refraction angles in the green were found to be approximately *±*60° measured from the normal to the HPDLC film surface. Such wavelength dispersion diminished when E*_ex_* was larger than 9 V/*μ*m, confirming the electrical control of the 2D honeycomb-like PhC using an HPDLC. We note that the wavelength dispersion as shown in Figure 12 was observed neither by normally incident p-polarized white light nor from a 2D honeycomb-like PhC fabricated by three p-polarized plane-wave interference holographic polymerization. It is also interesting to note that no wavelength dispersion was observed when a similar 2D PhC structure was made without ITO electrodes.

**Figure 13.**
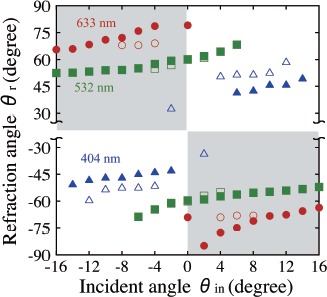
Refraction angles as a function of incident angle at different wavelengths and at s polarization. Measured and calculated data points are denoted by (▲, ■, •) and (△, □, ○) at (404 nm, 532 nm, 633 nm), respectively. The white and gray regions denote positive and negative refraction, respectively.

In order to quantitatively investigate effects of the observed wavelength dispersion phenomenon on incident ray angle and wavelength, we measured dependences of refraction ray angle on incident ray angle at different s-polarized laser wavelengths (404, 532 and 633 nm) for the same HPDLC film as used in Figure 12. The result is shown in Figure 13, where the incident ray angle *θ_in_* (the refraction ray angle *θ_r_*) is defined as an angle between the surface normal of the HPDLC film’s substrate and the incident (refracted) ray in the counter-clockwise direction. Both incident and refraction angles have the same (opposite) signs when the positive (negative) refraction takes place. Calculated results by the FDTD analysis are also plotted in Figure 13. It can be seen that, as similar to the observation by Li *et al.* [34], strong wavelength dispersion occurs at three wavelengths. While only the positive refraction was observed at 404 nm in our experiment, the calculation predicted the peculiar negative refraction at *θ_in_* = *±*2° (see two open triangle symbols in the hatched areas in Figure 13). We also observed both positive and negative refraction at 532 nm in the incident angle range of *−*6° ≤ *θ_in_* ≤ +6° and also at 633 nm with *θ_in_* = 0°. Furthermore, we only observed the negative refraction at 532 nm in the incident angle range of *|θ_in_| ≥* 6° and at 633 nm with *θ_in_* ≠ 0°. Although these observations could be explained partly by our numerical analysis, further investigation is necessary to clarify the discrepancy between measured and calculated results.

## 5. Conclusions

We have studied the holographic fabrication of 2D PhC structures in HPDLC films and their electrically controllable optical properties both theoretically and experimentally. We have employed the 2 *×* 2 matrix formulation and the statistical thermodynamic model to calculate the refractive index distributions in HPDLC films by three-beam holographic polymerization. We have shown that refractive index distributions are strongly influenced by the polarization state of three waves and by multiple reflections between two transparent ITO electrodes that are used for electrical tuning of PhC structures. It has been found that while a 2D honeycomb-like PhC structure forms by three s-polarized plane-wave interference holographic polymerization, a triangular-like photonic lattice structure forms by three p-polarized plane-wave interference holographic polymerization. Their local lattice structures are significantly altered by the multiple interference effect. We have confirmed such a difference in PhC symmetry by the examination of SEM images of recorded HPDLC films. We have also observed a strong polarization dependence of the optical properties including spectral transmittance and wavelength dispersion. It has been found that an incident s wave propagating in the 2D honeycomb-like PhC structure exhibits the broadband wavelength dispersion from the blue to the red with the dispersion angle of *∼*25°. We has also shown that such a wavelength dispersion phenomenon gives the negative refraction under limited incident conditions. We have demonstrated that the observed wavelength dispersion can be completely suppressed by externally applied electric fields higher than 9 V*μ*m. Since our theoretical analysis relies on the statistical thermodynamic model that considers a single phase mixture of LC and the formed polymer for the photopolymerization-diffusion kinetics, more realistic and exact statistical thermodynamic models [62,63] that take the LC droplet formation and the nematic ordering process in the phase separation dynamics into account may explain our experimental results more accurately and answer to our unsolved questions on anisotropic spectral transmittances and negative refraction. Moreover, it would also be interesting to investigate possibilities of novel optical phenomena and their electrical control of 2D HPDLC honeycomb (graphene)-type photonic lattices in linear and nonlinear optical regimes.
